# ID 64 - Revisão Sistemática de Modelos e *Frameworks* para Avaliar a Implementação de Guias de Práticas Clínicas: análise de domínios

**DOI:** 10.5327/2237-9622.2025.v34s1.64

**Published:** 2025-11-25

**Authors:** Nicole Freitas de Mello, Sarah Nascimento Silva, Dalila Fernandes Gomes, Juliana da Motta Girardi, Jorge Otávio Maia Barreto

**Keywords:** guias de prática clínica, ciência da implementação, Sistema Único de Saúde, modelos teóricos

## Abstract

**Introdução:**

Guias de práticas clínicas (GPC) orientam o cuidado mais adequado frente às melhores evidências disponíveis e são a base para o gerenciamento da utilização de tecnologias em um sistema de saúde. Para alcançar os resultados e os impactos desejados, os GPC devem ser adequadamente implementados, o que exige planejamento cuidadoso, monitoramento contínuo e a colaboração de várias partes interessadas. O uso de modelos e (M&F) pode apoiar a avaliação da implementação, permitindo maior estruturação e padronização desse processo. Considerando a lacuna de conhecimento sobre M&F disponíveis para avaliar a implementação de GPC, este trabalho teve como objetivo mapear M&F utilizados para essa finalidade e analisar seus domínios avaliativos.

**Método:**

Uma revisão sistemática foi conduzida seguindo a metodologia Cochrane, relatada pelas diretrizes PRISMA e registrado seu protocolo no PROSPERO (CRD42022335884). As buscas foram conduzidas até a data de 15 de maio de 2023, em 14 bases de dados e complementadas por buscas manuais. Utilizou-se o descritor, além dos sinônimos e dos termos livres, , e . A seleção e a extração de dados foi realizada em duplicata, por revisores independentes. Todos os documentos que reportaram o uso de M&F para avaliar a implementação de CPG foram incluídos. Os domínios dos M&F foram analisados de acordo com os constructos de Nilsen adaptados: "intervenção", "estratégias", "contexto" (micro, meso, macro ou múltiplo), "resultados" (aceitabilidade, adequação, viabilidade, adoção, custo e penetração), "fidelidade e adaptação" e "sustentabilidade". Foram utilizadas as ferramentas da JBI para avaliação crítica da confiabilidade, relevância e resultados dos estudos selecionados.

**Resultados:**

Todos os M&F abordaram o domínio "contexto" (100%), com destaque para o nível micro (66,7%), que traduz os aspectos individuais que afetam a implementação dos GPC, seguido por múltiplos níveis (58,3%), nível meso ou organizacional (33,3%) e nível macro ou estrutural (16,7%). O domínio "desfecho" foi avaliado em 75% dos M&F, com os seguintes subdomínios abordados: "adoção" (50%), "aceitabilidade" (33,3%), "adequação" (25,0%), "viabilidade" (25,0%), "custo" (8,3%) e "penetração" (8,3%). Domínios como "intervenção" (66,7%), "estratégias" (58,3%) e "processo" (41,7%) também foram frequentemente abordados. Em contrapartida, o domínio "sustentabilidade" foi pouco encontrado (8,3%), e "fidelidade/adaptação" não foram observados.

**Conclusão:**

Os M&F apresentaram domínios analíticos variados, com conceitos às vezes divergentes. Observou-se uma personalização dos domínios de análise, onde alguns domínios propostos pelos M&F não foram utilizados, enquanto outros foram incluídos de forma complementar. Isso reforça a ideia de que os M&F devem ser utilizados como guias para o processo avaliativo, e não como instrumentos rígidos com requisitos obrigatórios.

A aplicação de M&F na avaliação da implementação de GPC contribui diretamente para melhores impactos sociais da Avaliação de Tecnologias em Saúde (ATS). Este estudo é inovador ao enfatizar o uso de M&F para o estágio de avaliação da implementação de GPC e ao resumir aspectos e domínios voltados para sua aplicação prática, podendo subsidiar o debate e as escolhas de M&F para as práticas avaliativas em sistemas de saúde e contribuir para uma gestão de tecnologias eficiente.

